# Navigating ethical challenges in donation after circulatory death: Nurses’ perspectives

**DOI:** 10.1177/09697330261428608

**Published:** 2026-03-10

**Authors:** Emilie Gripewall, Janet Mattsson, Lisbeth Fagerström, Gunilla Björling, Linda Estman

**Affiliations:** 1 Faculty of Science and Engineering, Department of Natural and Health Sciences, 1040 Åbo Akademi University, Vaasa, Finland; 2 National Donation Center, National Board of Health and Welfare, Stockholm, Sweden; 3 Department of Health Science, Linneus University, Växjö, Sweden; 4 Faculty of Health and Social Sciences, University of South-Eastern Norway, Borre, Norway; 5 Department of Neurobiology, Care Sciences and Society, Karolinska Institute, Stockholm, Sweden; 6 Department of Nursing, School of Health and Welfare, 145651 Jönköping University, Jönköping, Sweden; 7 School of Nursing, KCMC University, Moshi, Tanzania; 8 Department of Anaesthesia and Intensive Care, Södertälje Hospital, Södertälje, Sweden; 9 Department of Caring and Ethics, 56627 University of Stavanger, Stavanger, Norway

**Keywords:** organ donation, donation after circulatory death, caring ethics, critical Incident technique, intensive care nursing

## Abstract

**Background:**

Controlled Donation after Circulatory Death (cDCD) offers new opportunities but also new, demanding ethical challenges in the field of organ donation. The process requires navigating boundaries due to the transition from intensive care and life-sustaining treatment to the end-of-life care and a possible cDCD process. Previous research has mainly focused on medical aspects, leaving a gap regarding intensive care nurses’ and their reflections of possible, ethical challenges during the cDCD process.

**Aim:**

To explore ethical challenges intensive care nurses encounter when navigating life-sustaining treatment, its withdrawal, and potential organ donation in cDCD.

**Research design:**

A qualitative design with an inductive approach was applied using Critical Incident Technique.

**Participants and research context:**

The study was conducted at two Swedish ICUs and included multiple scenarios when cDCD was managed after withdrawal of life-sustaining treatment. Five experienced intensive care nurses were interviewed, yielding 142 critical incidents for analysis.

**Ethical considerations:**

The study followed the Declaration of Helsinki and Swedish law on ethical vetting. Approval was obtained from the Swedish Ethical Review Authority (ref. 2017/1722-31-1).

**Results:**

Five themes were identified: (1) Navigating Ethical Boundaries in End-of-Life Care and cDCD, (2) Safeguarding Autonomy in cDCD Decisions, (3) Balancing Emotional Presence and Ethical Practice, (4) Managing Ethical Uncertainty Through Knowledge and Team Support, and (5) Ensuring Ethical Responsibility Through Interprofessional Communication. Ethical challenges were linked to unclear procedures, limited training, and fragmented communication, while structured guidelines and collegial support enabled ethical coherence.

**Conclusions:**

cDCD requires continuous negotiation between ethical-, professional-, and emotional dimensions. To ensure ethically sustainable practice, organisations need to strengthen ethical awareness, structured training, and interprofessional communication. Integrating both biomedical principles and caring ethics is crucial to safeguard patient autonomy, support relatives, and enabling healthcare professionals to act with confidence during end-of-life care and organ donation.

## Introduction

Donation after Circulatory Death (DCD) has emerged as an important strategy to address the shortage of organs for transplantation across Europe during the last year.^1^ Depending on counties’ different legislation, DCD could be a controlled (cDCD) or uncontrolled (uDCD) process.^2,3^ In Sweden, controlled DCD (cDCD) was implemented in 2020 and increased the number of transplantable organs.^4^ However, cDCD introduces new possibilities as well as significant ethical challenges.^5,6^ Ethical challenges may arise when nurses encounter value conflicts or uncertainty regarding the ethically appropriate course of action.^7^ Although, determination of death by circulatory criteria influences novel technologies, techniques, and therapies, that may generate ethical challenges before, during, and after the process.^5,8,9^ cDCD, in relation to withdrawal life-sustaining treatment, may lead to ethical challenges, particularly when it comes to the possibility to involve the patient, during stage of end-of-life-care, in the discussion about their willingness to donate organs after death.

Critical incidents are described as concrete events in which professional duties, patient interests, or legal obligations come into sharp tension, often requiring immediate and ethically charged responses.^10^ Identifying such situations that significantly affect ethical outcomes from the intensive care nurses’ perspective will be essential for understanding ethical dimensions of the cDCD process.^11^ For intensive care nurses, cDCD involves navigating ethical boundaries between continuing life-sustaining treatment, withdrawing treatment, and considering the possibility of organ donation. There is a notable empirical gap in published evidence regarding ethical experiences from intensive care nurses’ perspective of cDCD, particularly from a caring ethics perspective. Studying both positive and negative critical incidents that give rise to ethical challenges can therefore deepen our understanding of how nurses navigate these complex situations.^10,12^

## Background

When care changes, from life-sustaining to a withdrawal, the question regarding patient’s willingness to donate organs after death should be integrated into intensive care practice.^1^ Organ donation, whether from living or deceased donors, must always respect human dignity^13^ and the individual’s right to make autonomous decisions regarding their own body.^14^ Understanding the four fundamental ethical principles – beneficence, nonmaleficence, autonomy, and justice – is essential in all healthcare contexts.^15^ To reduce ethical confusion and prevent ethical barriers related to cDCD, healthcare professionals require knowledge of potential ethical challenges and how these may emerge in practice.^5,8^ In addition, caring ethics, as conceptualised in Eriksson’s caritative caring theory, emphasises respect for human dignity as the foundation of ethical decision-making.^13,16^ This perspective highlights that beyond ethical principles, healthcare practice must be grounded in compassion and a genuine commitment to alleviate suffering, thereby providing a complementary lens for understanding the ethical challenges associated with cDCD.

Unlike Donation after Brain Death (DBD), when death is determined by neurological criteria after, for example, catastrophic brain injury,^17^ DCD opens for other patient groups possibility to donate their organs after death.^9^ Depending on the patient ongoing, life-sustaining treatment, willingness and decision-making may involve the possibility to interact and involve the patient when treatment no longer benefits the patients’ life. Engaging in conversations about withdrawal treatment and organ donation at this stage can be ethically demanding.^18^ Healthcare professionals, particularly bedside nurses, must therefore be prepared to navigate this transition, to balance compassion, autonomy, and professional responsibility.^19^

In situations when question about organ donation is raised in connection with termination of life-saving treatment, ethical challenges may arise. When a patient receiving Extracorporeal Membrane Oxygenation (ECMO)-support, this transition becomes more ethically complex. ECMO provides temporary support for the heart and/or lungs when they are unable to function adequately.^20^ The meaning of ECMO is to give a critically ill patient time to recover from life-threatening conditions. Patients with ECMO-support will preferably be awake and communicable, as this has been associated with improved survival rates and fewer complications compared to sedated or unresponsive patients, despite their critical condition.^21^ However, such awareness also opens the possibility for patient involvement in discussions about withdrawal and donation, intensifying ethical questions around informed consent, willingness, and therapeutic misconception.^5^

Ethical considerations and awareness of potential ethical challenges associated with cDCD have increasingly been discussed in recent years in the international context.^3,6^ Possible confusions, doubts, and ethical challenges must be addressed in early stage, otherwise the donor process may be compromised.^3,6,22^ Critical incidents concerning challenges in defining the break-point decision and the transition from life-saving treatment to treatment for someone else, as well as thoughts about when the patient is dead, warrant further investigations.^3^ A broader understanding of these issues could complement existing research and provide guidance in ethically complex cDCD cases.^3,8,18,19,22^ Also, the establishment of clear boundaries and limitations for the healthcare team in the cDCD process may help to reduce ethical challenges related to cDCD at units with additional interventions, such as ECMO-support, and will therefore be of an interest to study.^8,9^

Clear national regulations and guidelines governing end-of-life care and cDCD are central to maintaining public trust and supporting ethically consistent practice among healthcare professionals.^1,5,18^ Thus, there is a significant knowledge gap regarding nurses’ perspectives on ethical challenges in cDCD, particularly regarding establishing clear boundaries in the cDCD process. Exploring critical incidents from intensive care nurses’ perspective can therefore provide valuable insights for ethically sustainable practice and a deeper understanding of this evolving field.

## Aim

The aim of this study was to explore ethical challenges intensive care nurses encounter when navigating life-sustaining treatment, its withdrawal, and potential organ donation in cDCD.

## Method

### Design

**Figure 1.**
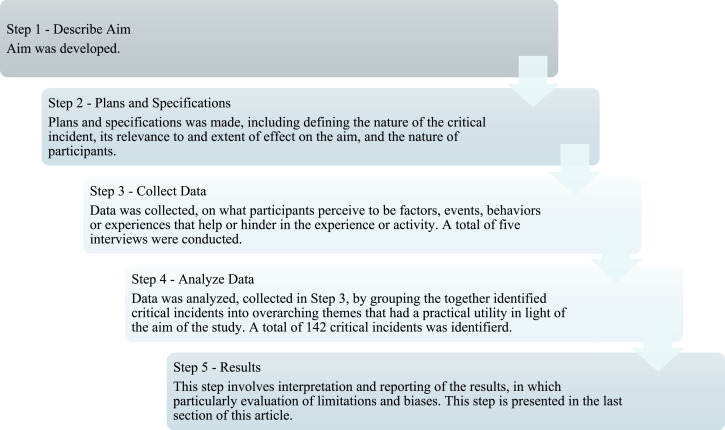
Overview process, step-by-step.

This study has a qualitative research design with an inductive approach. Critical Incident Technique (CIT), originally developed by Flanagan^12^ and later refined by Tombs^10^ and Viergever,^23^ was used as methodological framework. The method provides a systematic step-by-step process for identifying, documenting, analysing, and learning from both negative and positive incidents, with the aim of improving future practice and preventing similar occurrences^10,12^. In this study, we followed Viergever’s^23^ structured step-by-step approach (Figure 1).

### Research question

In the first step, a research question was formulated to address the study aim:

*What ethical challenges do intensive care nurses express, when navigating life-sustaining treatment, its withdrawal, and potential organ donation in the context of cDCD?*

## Settings and participants

Step two involved planning and specifying how to gather critical incidents to meet the study aim.^11^ Two intensive care units (ICUs) in Sweden were selected based on their experience with clinically challenging cDCD processes following the withdrawal of life-sustaining treatment. Multiple cDCD scenarios, each significantly affecting the course or outcome of care, were included in the study. Inclusion criteria were registered ICU nurses with experience of the selected cDCD cases, clinical experience, and prior knowledge of organ donation through the DBD-process, but without previous formal education, training, or experience in DCD. Exclusion criteria were other healthcare professionals, ICU nurses not directly involved in the specific cDCD processes, and nurses with prior formal DCD education, training, or experience. All registered ICU nurses who had been directly involved in one or more of these scenarios were identified and invited to participate. A total of eight nurses were invited, and five agreed to participate.

All participating nurses met the inclusion criteria. To protect patient and staff confidentiality, no additional background variables are reported.

### Data collection

Data were collected through interviews focussing on the nurses’ experience regarding selected scenarios. cDCD processes are rare events, and the interviews were therefore conducted between 2021 and 2023. Each interview was approximately 50 min long. The participants were contacted by two of the authors (NN and NN) to schedule interview times.

The first interview was conducted as a semi-structured dyad interview with two participants, while the remaining three were individual in-depth interviews. This design allowed for both shared reflection and deeper individual exploration of the phenomenon. All interviews began with the open-ended question: *‘Can you tell me about your experiences of this specific case (cDCD)?’* Follow-up questions addressed the participants’ previous experiences with organ donation. The first interview was conducted by author (NN), and the remaining three by author (NN). All interviews were audio-recorded and transcribed verbatim by author (NN).

## Data analysis

**Figure 2.**
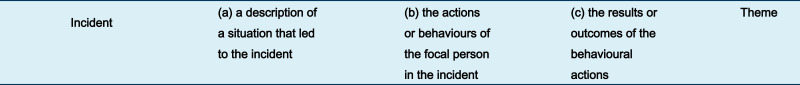
Model of structure CIT table used in the analysis.

Data was analysed using Tombs’^10^ three-step CIT analytical approach. In the first step, all transcript data relevant to the research question were identified and organised into tables structured according to the CIT format^10^ (Figure 2).

A total of 142 critical incidents regarding ethical tensions were identified and entered (Figure 2). All incidents were initially categorised into several broad thematic categories. In the second step, the categories were compared for similarities and differences and then reorganised and clustered into five overarching themes by authors (NN, NN, and NN). In the third and final step, the thematic structure was cross-checked through collaborative discussions among all authors to ensure consistency, credibility, and trustworthiness of the categorisation process and the resulting themes.^10^ The final analysis resulted in five main themes, which are presented in the result section.

### Ethical considerations

This study was conducted by following the ethical principles established by the Declaration of Helsinki^24^ and Swedish law for ethical vetting (2003:460). Ethical approval was obtained from the Swedish Ethical Review Authority (reference number: 2017/1722-31-1) as a part of a larger research project. The participants provided informed consent and could withdraw from the study at any time.

## Results

The critical incidents highlight that nurses’ experiences of cDCD involve challenges related to ethical-, professional-, and emotional dimensions. Five themes were identified^1^: Navigating Ethical Boundaries in the End-of-Life Care and cDCD,^2^ Safeguarding Autonomy in cDCD Decisions,^3^ Balancing Emotional Presence and Ethical Practice,^4^ Managing Ethical Uncertainty Through Knowledge and Team Support, and^5^ Ensuring Ethical Responsibility Through Interprofessional Communication.

### Navigating Ethical Boundaries in the End-of-Life Care and cDCD

A recurring theme was the need for clear boundaries, physical, clinical, and medical, to state the start of a new phase, and the transition from lifesaving treatment into a cDCD process. One boundary was seen when lifesaving ECMO-support was disconnected and replaced by another similar machine from the transplant team, maintained to optimise the organs only for transplantation after the patient was declared dead by circulatory criteria. This technical step signified a profound ethical boundary shift: ‘*We’re letting go, we’re cutting off our tubes here. Now they’re taking over’ (C)*. Such moments underscored the tension between continuing care for survival and facilitating organ donation, a tension experienced not only as a clinical transition but as an existential and ethical threshold. This action was an example of how a medical procedure also took on a function, as a confirmation that the care aimed at survival had now ended and that a new responsibility had been taken over by another team. This threshold was pivotal for the nurses as the transition from life to death became evident, from both medical and ethical perspectives.

Another boundary described concerned how the professional role was affected by proximity to the patient and family. One nurse narrated the relief of not having cared for the patient before the donation process, which made it easier to maintain a professional distance. This reflection highlights the necessity to ‘take a step back’, not as a form of evasion, but as an active boundary-setting, to protect both the own dignity and the patient. In contrast, it was described how some patients ‘come home with you’, an expression of how emotional work does not always end at the same moment as the treatment, or when finishing the working shift. Nurses also described how the proximity of death requires a shift in the focus of care, from motivating to enabling a dignified end. This shift conveys practical step, intertwined with an existential act in the caring process. The nurses expressed that when the patient is still awake and conscious, the sense of responsibility becomes greater: ‘*The last hours should be as good as possible*’ *(B)*. This conveys nurses’ dual responsibilities, partly towards the dying patient and towards the transition to caring for someone else’s survival through donation.

It emerged that the uncertainty surrounding when death occur could create internal conflicts in the healthcare team. In one situation, the moment of donation was almost perceived as a competition, that the patient had to die ‘in time’ for the organs to be usable. This feeling caused ethical stress and discomfort but also served a possibility for reflection and reminders about how important it is to preserve dignity and understanding of the boundary of death, even when time and technology put pressure on the healthcare staff.

### Safeguarding Autonomy in cDCD Decisions

This theme highlights how nurses experience ethical tensions when organisational structures, professional insecurity, and emotional discomfort overshadow patients’ right to self-determination in cDCD. Although the principle of autonomy is a cornerstone in all healthcare ethics, critical incidents revealed how cDCD decisions were sometimes influenced more by healthcare professionals’ personal emotions and uncertainty than by the patient’s expressed or presumed wishes.

One challenge occurred when a patient, registered and willing as an organ donor, was not donated due to unclear procedures. *‘They messed it up completely, so unfortunately it didn’t happen’ (A)*. This case highlights an ethical tension; healthcare professionals experienced a conflict between respecting the patient’s documented autonomous choice and managing their own uncertainty and procedural ambiguity.

Another recurring issue was the inconsistent willingness of physicians to initiate organ donation discussions during the stage of decision to discontinue life-sustaining treatment. *‘It depends a little on which physician is on duty’ (D)*, suggesting that the patient’s autonomy is conditioned by the healthcare teams’ willingness to act. This contradicts the principle that every person has the right to make decisions about their body, even after death. When the conversation does not take place, autonomy is negotiated in silence, meaning that the patient’s autonomy could be undermined by organisational culture or individual preference.

In another challenge, a conscious patient being treated with ECMO-support, while a decision was made to withdrawal life-sustaining treatment. Nurses expressed an uncomfortable feeling to wake the patient up; told them the life-sustaining treatment will be removed and then ask about the willingness of organ donation: ‘*You don’t wake someone up in intensive care and tell them we’re going to turn off the machines, you just don’t do that’ (B)*. Despite this, importance of not withholding information about one’s own death was reflected: *‘It’s their body, their life’ (A)*. This highlights an ethical stance emerges in which respect for the other person’s subjectivity and right to participation outweighs the caregiver’s need for emotional security.

A recurring frustration among when responsibility for raising the issue of donation tended to fall on individuals was described: *‘It shouldn’t depend on who is working that day’ (A)*. This illustrates an organisational gap, where respecting autonomy becomes a personal rather than collective professional responsibility especially in situations where the patient can no longer speak for themselves.

### Balancing emotional presence and ethical practice

This theme highlights an ethical awareness among ICU nurse, where emotional presence is not seen as a weakness, but as a natural and sometimes essential for delivering dignified end-of-life care in the context of cDCD, and how emotional presence is integrated into ethical practice. The transition from intensive care to a cDCD process is not only a medical shift but also a deeply human responsibility, where nurses show caring in various ways, both for the patient and for their relatives. One example is striving to make this moment meaningful despite strong emotions. *‘My defence mechanism in all this is that I am so committed to making it a beautiful moment’ (B)*. This desire, to create a dignified ending, was helpful to stay present. Ethical stance was then expressed through an active choice to not withdraw. Instead, compassion could be used as a tool to address suffering. Emotional presence such as grief, tears, and touch is allowed to take place, and it is not perceived as an obstacle to professionalism but as expression of human dignity.

Nurses also emphasised the importance of ethical vigilance when technical or procedural deviations occurred, prompted reflections on how these factors affect relatives’ experiences of farewell. They reflect how this affected the experience of relatives saying goodbye and emphasise the importance of clarity, respect, and adhering to procedures based on both law and ethics: *‘This turned out differently… it’s important that we do as we usually do’ (C)*. Although the experiences are often emotionally charged, the participants also express a willingness to remain in the difficult situation rather than avoid it.

### Managing Ethical Uncertainty Through Knowledge and Team Support

This theme highlights how clinical complexity, ethical uncertainty, and emotional strain in cDCD are influenced by the availability of knowledge, clear procedures, and team support. When these elements were missing, nurses described uncertainty about legal frameworks, procedural steps, and communication with relatives, prerequisite for ethical action.

Several emphasise the importance of collegial presence and access to reflection, not merely as a way of ‘managing’ emotions but as an opportunity to create meaning together in what is happening. One nurse describes it this way: *‘I don’t think it’s hard to cry… it’s a way for me to flush things out a bit’ (B)*. This openness to emotions creates space for an ethical practice where staff both bear and share the difficult, rather than being isolated in it. The nurses also describe the need for reflection, support from colleagues, and the opportunity to verbalise their experiences. Without debriefing, a risk of carrying emotional residue from previous events was described, which affects both the working environment and future decision-making.

The lack of knowledge shifted, from evidence-based practice to assumptions and subjective interpretations, which in some cases leads to decisions being made on uncertain grounds. The fact that the cDCD process was carried out in certain situations without all staff involved understanding its meaning reinforces the need for a common knowledge base and interprofessional training initiatives. Several respondents describe how lack of training created uncertainty about law, procedures, and communication. Lack of knowledge opens doors for speculation, which in turn contributes to a feeling of disagreement and moral doubt: *‘Are we in a grey area now, or did we act in accordance with the law?’ (C)*.

Conversely, when teams had access to checklists, guidelines, and experienced mentors, the process was described as safe and well-coordinated. In these cases, the medical complexity is perceived as manageable and the ethical uncertainty as something that can be discussed openly in the team: *‘The checklists were incredibly good’ (E)*. Upcoming challenge shows that these events are not only significant for the individual care situation but also for organisational learning. When experiences are not utilised or shared, important knowledge is lost and future situations risk being met with the same uncertainty as before. A climate where the exchange of experiences is hampered by hierarchical structures or a culture of silence hinders the implementation of new working methods, which in the long run can lead to potential donors not being identified: *‘I was there the whole time, but then I was told that no, you shouldn’t talk about that’ (D)*.

### Ensuring Ethical Responsibility Through Interprofessional Communication

This theme reveals how breakdowns in interprofessional communication affect both the healthcare team and the relatives during the cDCD process. Nurses described recurring communication breakdowns during crucial decisions. This is particularly evident in connection with withdrawal conversations, the termination of life-sustaining treatment, and donation processes. The nurses describe how crucial conversations between physicians’ and relatives often take place without other staff being informed, which means that nurses and nursing assistants are unable to meet the parents with an understanding of what has been said. This not only creates uncertainty for the healthcare team but also risks undermining the caring conversation as a place for support, participation, and mutual understanding, and affects the ability to maintain a caring conversation characterised by community, continuity, and security.

It was described how the physicians sometimes conduct turning point conversations with relatives without informing the rest of the team, especially the nurses, about the content of the conversation: *‘The parents come in, and we don’t even know that it has happened. It becomes very strange’ (A)*. This caused confusion and inability to adequately support families, and created a feeling of exclusion, uncertainty, and ethical stress, especially since it was often the nurses who met with the families immediately afterwards. The nurses testified to difficulties in responding to questions, addressing concerns and providing adequate support when they were unaware of the content of the decision or what had been said: *‘The parents had been told that there was nothing more that could be done, but I didn’t know that. I didn’t understand why they were so distraught’ (D)*.

The lack of joint feedback after such conversations was seen as an obstacle to the nurse’s ability to stand united before the relatives and to jointly bear the ethical responsibility that comes with difficult news. Instead of a cohesive care team, there was a sense of fragmentation, with different professions acting on different information. This undermined the potential of the caring conversation to create security, both for the patient’s relatives and for the staff themselves: ‘*We need to know the same thing. That’s the only way to give parents security, that we stand united’ (A)*.

The nurses also emphasised that caring conversations is not limited to a specific occasion but is an ongoing process that requires consistency and trust within the team. When the conversation about the focus of care is not shared, the care itself is also perceived as fragmented. This gave rise to ethical stress, where nurses felt that they could not take responsibility in a morally sustainable way because they were not involved in the decision-making process: *‘When we are not allowed to participate in the conversation, we are also deprived of responsibility. But we are the ones who must stay in the ward, explain, interpret and comfort’ (B)*. Several also pointed out that this shows a lack of respect for the team’s collective expertise and for the ethical importance of continuity in communication. The psychological security led to an ethical safety within the team, and some described how this created a silence or hesitation to raise their own thoughts.

Lack of consensus was also manifested at organisational level, that manifested and interpreted ethical challenges when different bodies within the organisation speak in different languages complaining a risk of interpreting concepts and guidelines differently. This becomes particularly clear in situations where conceptual confusion arises, for example, between ECMO-treatment and Normothermic Regional Perfusion (NRP), as well as in the concept of brain death and definitions surrounding DBD and DCD. These misunderstandings risk leading to incorrect decisions or ethically questionable actions.

Nurses highlighted lack of joint reflection and debriefing after demanding care situations as a complicating factor. Despite obvious needs, there were few structured opportunities to share experiences, feelings, and ethical dilemmas. This hinders learning and development within the team and contributes to important experiences not being utilised. At the same time, when communication works, for example, in the form of close dialogue with the transplant coordinators, the process is perceived as safe, professional, and cohesive.

## Discussion

The five themes in this study show that cDCD is not merely technical but deeply ethical, involving boundaries, autonomy, emotions, knowledge, and communication.

The first theme highlighted the ethical boundaries between life, death, and the professional role. While previous research has described cDCD as an ethically sensitive process,^9,18^ this study complements this by showing how technical procedures, such as the disconnection of ECMO and the establishment of a clear boundary between life-sustaining treatment and the transition to a cDCD process, acquire more than symbolic meaning as markers of ethical thresholds. Nurses’ ethical dilemmas must also be understood in relation to the social and cultural context of intensive care.^7^ This practice is shaped by a strong rescue-oriented culture, where saving life, sustaining hope, and mobilising advanced technology are normative expectations that shape professional identity and practice.^18^ This socialisation influences how nurses perceive ethically complex situations,^7^ such as the transition from life-sustaining treatment to cDCD, which may be experienced not only as a clinical shift but as a disruption of the prevailing ethical narrative of intensive care. Boundaries are also moral and existential, aligning with studies emphasising the importance of dignity and ethical presence in intensive care.^3,13,16^ Nurses actively manage emotional distance as an ethical act, not avoidance. Therefore, clarity, reflection, and collegial support were seen as essential to safeguard the dignity of patients, relatives, and staff, echoing the recommendations for structured guidance in cDCD processes.^1,5,6^

The second theme showed that patient autonomy was challenged by organisational routines, procedural ambiguity, and individual hesitancy. Respect for patient autonomy is a cornerstone of healthcare ethics^3,15^ and the patients’ right to be treated as a unique human being, with respected human values, must be pursued even in complex situations.^13^ According to Eriksson, autonomy is inseparable from dignity. Upholding autonomy thus becomes an ethical act of recognising the patient’s humanity and dignity, even in complex transitions such as cDCD.^13,16^ However, the critical incidents revealed how organisational culture, lack of procedures, and personal insecurity sometimes overshadowed patients’ right to self-determination in organ donation. Although the prevalence of such uncertainty cannot be determined, the findings indicate that insufficient knowledge and communication could, in the worst case, contribute to failure in donor identification or interruption of the donation process. This finding supports previous observations that respecting autonomy and dignity requires more than legal knowledge.^3,13^ It demands ethical sensitivity, active listening, and prioritising the patient’s perspective over professionals’ convenience or uncertainty.^8,15,18^ Nurses’ accounts of silenced or overlooked patients’ wishes reflect broader ethical discussions on the risks of undermining autonomy and dignity in cDCD contexts.^3,6,13^

The third theme addressed the emotional impact of cDCD. Emotional presence was viewed, not as a weakness but as an essential aspect of ethical practice, where compassion and vulnerability sustain dignified end-of-life care. In line with Eriksson’s theory,^13,16^ emotional sensitivity is understood as an ethical response rather than personal fragility, reflecting nurses’ moral engagement with patients’ suffering. By remaining emotionally present, nurses affirm the patient’s dignity and preserve the ethical meaning of caring, even in close proximity to death.

The fourth theme underscores the importance of knowledge and team support as prerequisites for ethical action. When absent, uncertainty and moral doubt prevailed, whereas structured guidelines and mentorship facilitated confidence and coherent practice.^1,3,19,21^ Ethical challenges in cDCD thus appear to be systemic rather than purely individual, requiring collective learning, standardised protocols, structured debriefing, and a culture that value interprofessional dialogue.^3,5,8^

The fifth theme states that interprofessional communication emerged as central ethical imperatives and responsibility. Breakdowns in interprofessional dialogue risked undermining both staff confidence and families’ experiences of security, reflecting similar findings on the relational and organisational dimensions of intensive care.^13,18^ Structured opportunities for joint discussion, a common knowledge base, and a clear division of responsibilities were highlighted as critical for ensuring cohesive, ethically sound care.^6,9^ Lack of communication and team consensus emerged as recurring sources of ethical stress.^25^ When critical conversations with relatives were held without the involvement of the whole team, nurses felt excluded, unable to support families adequately, and deprived of shared responsibility.

These themes illustrate that cDCD is not only a clinical and technical process but also a deeply ethical and relational challenge. Nurses’ testimonies reveal how boundaries, autonomy, emotions, knowledge, and communication are interwoven, shaping both the care of the dying patient and the transition to organ donation. While biomedical ethical principles remain essential, they must be complemented by a caring ethics perspective. Drawing on Eriksson’s caritative caring theory,^13,16^ ethical practice can be understood as grounded in *caritas*, the ethical will to alleviate suffering and protect human dignity. Nurses’ emphasis on compassion and relational presence reflects this understanding, where ethics is expressed through genuine caring rather than procedural compliance. This aligns with previous literature highlighting the need for ethical sensitivity, structured guidance, and relational presence to ensure clarity and security in complex cDCD processes.^1,3,15,18^

### Limitations and strengths

The scenarios involved complex clinical challenges and were therefore well suited to examination as critical incidents in relation to the study’s aim. The use of CIT enabled an in-depth exploration of ethically significant situations from nurses’ perspectives, allowing both positive and negative experiences to be identified.^10,12^ In accordance with Lincoln and Guba’s^26^ criteria for trustworthiness, the application of CIT strengthened the credibility of the findings by grounding interpretations in rich, context-specific narratives. Thus, the limited number of participants at two ICUs in Sweden limited the generalisability and may restrict the transferability of the results, although diversity and depth of data that covering all lifespans enhance their potential relevance to other settings where cDCD is practiced. Although informed by CIT, the analysis focused on thematic interpretation rather than systematic incident–action–outcome mapping. Behavioural outcomes were therefore inferred rather than explicitly structured, which may be considered a limitation from a traditional CIT perspective. Moreover, the recurrence of core themes across incidents supports the dependability of the findings and indicates that they reflect broader ethical challenges within intensive care practice.

## Implications for practice and future research

The findings suggest that proximity and continuity in nurse–patient relationships may influence ethical experiences during cDCD, and therefore point to several important implications for clinical practice. First, there is a need for training and continuous education on cDCD, including knowledge of legal frameworks, ethical considerations, and communication strategies. There is also a need for actively training, strengthening ethical competence, and communication skills related to the cDCD process, for example, through formalised education and training programmes. Second, organisations should implement clear guidelines and provide opportunities for structured debriefing and collegial reflection, enabling the processing of emotional and ethical challenges in a supportive environment. Third, improving interprofessional communication and fostering team consensus are crucial to safeguard patient autonomy, strengthen family support, and reduce ethical stress.

Future research should further explore how interprofessional teams can best be supported in ethically complex end-of-life contexts, with particular attention to strategies that promote collaborative decision-making and moral resilience, including educational interventions aimed at enhancing ethical competence, to contribute to a broader understanding of how organisational structures shape ethical practice in cDCD.

## Conclusions

This study demonstrates that nurses’ reflections on cDCD are shaped by a continuous negotiation between ethical, professional, and emotional dimensions. The findings highlight that the cDCD process is not only medical and technical but also equally existential and relational, where boundaries between life and death, patient autonomy, and the integration of emotional presence are constantly at stake. While insufficient training may contribute to uncertainty in autonomy-related decisions, the findings indicate that ethical challenges to patient autonomy are also shaped by organisational structures, professional hierarchies, emotional burden, and the rescue-oriented culture of intensive care. Ethical challenges were also amplified by unclear procedures and fragmented communication, whereas guidelines, collegial support, and interprofessional consensus emerged as prerequisites for ethically sustainable practice.

Communication was indicated as a central ethical mechanism in cDCD. Creating opportunities for interprofessional training, joint reflection and structured, multiprofessional communication practice is essential to ensure that cDCD will be initiated by healthcare professionals acting with confidence and ethical coherence, while the patient’s autonomy and dignity are respected. This underlines the need to strengthen ethical awareness, interprofessional dialogue, and reflective practices in cDCD contexts and supports a recommendation that senior clinicians with advanced communication training and emotional preparedness manage more emotionally demanding conversations. By recognising all ethical and relational dimensions of cDCD, healthcare organisations must therefore foster conditions that support both high-quality end-of-life care and trustworthy cDCD processes.
